# Targeted delivery of ursolic acid and oleanolic acid to lungs in the form of an inhaler for the management of tuberculosis: Pharmacokinetic and toxicity assessment

**DOI:** 10.1371/journal.pone.0278103

**Published:** 2022-12-29

**Authors:** Vinay Saini, Sujit Kumar Debnath, Priyanka Maske, Vikas Dighe, Rohit Srivastava

**Affiliations:** 1 Nanobios Lab, Department of Biosciences and Bioengineering, Indian Institute of Technology Bombay, Mumbai, Maharashtra, India; 2 National Centre for Preclinical Reproductive and Genetic Toxicology, National Institute for Research in Reproductive Health, Mumbai, Maharashtra, India; Siksha O Anusandhan University School of Pharmaceutical Sciences, INDIA

## Abstract

**Introduction:**

Ursolic acid (UA) and oleanolic acid (OA) are triterpenoids. They are used to treat numerous diseases, including tuberculosis. Combinations of these drugs provide new insight into the management of tuberculosis. The major obstacle is the effective delivery of these drugs to the lungs, which are mainly affected due to *M*. *tuberculosis*. A metered-dose inhaler (MDI) was developed to address this issue containing UA and OA, followed by *in-vitro* and *in-vivo* evaluation.

**Methods:**

In the present study, MDI formulation was prepared by incorporating UA and OA at the dose level of 120 μg/ml in each actuation. *In-vitro* evaluation of this MDI formulation was performed to ensure its suitability to deliver UA and OA preciously. With prior approval of IAEC, a pharmacokinetic and acute inhalation toxicity study was conducted using MDI on Wistar rats.

**Results:**

The pharmacokinetic study showed an increased biological half-life of UA (9.23±0.104 h) and OA (8.93±0.166 h) in combination therapy. *In-vivo* toxicity study demonstrated no adverse effects on body weight and vital organs in the treatment group compared with the control group. Histopathology examination of these essential organs showed no abnormalities. Mild alternation in the biochemical and hematological parameters was observed. However, these alterations did not affect the overall health of the animals.

**Conclusion:**

The present study documents a detailed study for the safety and pharmacokinetics of UA and OA *in-vivo* for their advanced application in tuberculosis disease.

## Introduction

Tuberculosis (TB) has been one of the most deadly diseases since the rapid emergence of multidrug-resistant TB. The hour needs to establish newer strategies to combat this disease. Naturally occurring triterpenoids are present in most fruits and medicinal herbs. One of the significant features of herbal medicines is the mixture of components with similar properties to achieve the synergistic activity. Triterpenoids, like ursolic acid (UA) and oleanolic acid (OA), have gained much attention in the medicinal and scientific community due to their proven pharmacological effects. These compounds are isomers to each other and have demonstrated numerous biological activities, including hepatoprotective [1], anti-tumor [2], and anti-hypertensive [3]. These drugs have also been reported to possess antibacterial activity [4]. OA and UA used alone or in combination have been reported to possess a synergistic effect against tuberculosis using H_37_Rv and an MDR clinical isolate in a macrophage cell line [5]. The anti-tuberculosis activity of OA has been reported in the extract of the Peruvian plant *Clavija procera* (*Theophrastaceae*) [6], *Buddleja saliga* (*Buddlejaceae*), and *Leysera gnaphalodes* (*Asteraceae*) [7]. Therefore, assessing their safety potency using in-vivo toxicity studies is essential to consider their potential applications. The ethanolic extract of *Eriobotrya japonica* leaf consists of these drugs. This extract was tested for acute and subacute toxicity in ICR mice. No mortality or adverse clinical symptoms have been reported at 0.3 to 3.0 gm/kg body weight [8]. LD_50_ > 300 mg/kg has been reported in mice with subcutaneous exposure to these drugs mixture obtained from *Bouvardia ternifolia* extract [9]. A 90-day subchronic toxicity study was conducted with UA and demonstrated no mortality or other signs of toxicity in Han-Wistar rats [10]. The subcutaneous and oral administration of these drugs has a few drawbacks like poor penetration, wider distribution, poor availability of the drug in the lungs, and severe unwanted side effects. In contrast, aerosol therapy enables drug administration directly into the lung, mainly affected by *M*. *tuberculosis*. The drugs can be delivered to the lungs or trachea using a metered-dose inhaler (MDI) or a dry powder inhaler (DPI). In this given study, we have introduced a newer approach to administering UA and OA through the inhalation route using MDI to target the lungs. MDI was developed and assessed the pharmacokinetic parameters (with a dose level of 120 μg/ml each) by administering the drug directly to the lungs of Wistar rats. Afterward, an inhalation acute toxicity study was performed to check the influence of chronic exposure dose. This inhalation therapy of UA and OA can be considered as an adjuvant therapy in the treatment of tuberculosis.

## Materials and methods

### Chemicals

Ursolic acid, oleanolic acid, corn oil, methyl-tert-butyl ether (MTBE), and methanol were procured from Sigma-Aldrich, US. Citric acid was procured from Merck Millipore, India.

### Anti-tuberculosis activity

The MH-S (murine alveolar macrophages) (ATCC-CRL-2019) cell line was derived by SV40 transformation. This cell line was infected with the H_37_Rv strain of *Mycobacterium tuberculosis* (ATCC No. 25618). The MTS [3-(4,5-dimethylthiazol-2-yl)-5-(3-carboxymethoxyphenyl)-2-(4-sulfophenyl)-2H-tetrazolium] assay was performed to assess *in vitro* anti-tubercular activity in 7H9 media. The culture cells were seeded to optimum consistency. Afterward, 40 μl of MTS reagent was added to each well. The cells were further incubated and measured the OD at 490 nm. The antitubercular activity of combined drugs was compared with the cell treated with isoniazid.

### Formulation and characterization of metered-dose inhaler

#### Formulation of MDI

Hydrofluoroalkane (HFA) was used as a propellant to maintain the pressure inside the canister. This type of propellant is unlike chlorofluorocarbons. It does not contribute to the depletion of the stratospheric ozone layer and is inert in the human airway [11]. The required quantity of UA and OA was filled in a canister. Both these drugs were dissolved in the propellant. After placing the cap, this canister was filled with propellant. The sealed cans were sonicated in cold water (10°C) for 15 min. HFA was filled in the can to achieve the desired weight using the aerosol filling unit attached to a propellant storage cylinder [12].

#### Estimation of average weight per metered dose

The actuator nozzle is essential for the aerosol formation, as the propellant vapor is pulled separately to form dispersed liquid droplets by an aerodynamic force rendering aerosol formation of the drug. The inner orifice diameter was 0.5 mm to assist the release, whereas the outer diameter of the drug outlet was set at 5 mm with a 3 mm opening for the drug release. Propellant’s density inside the canister influence the shot weight. The testing of average weight per metered dose helps assess the ability of a metering valve to deliver the content inside in terms of weight. Initially, the weight of canisters and actuators was recorded. The canister’s weight was recorded after five tests fired in the air (W_1_). Afterward, five successive sprays were delivered again. Actuators and canisters separated each other and wiped the orifice. These canisters were reweighed (W_2_), and the average metered dose was calculated per weight = (W_1_-W_2_)/5. This test was performed in triplicate to check the reproducibility.

#### Rate of evaporation, number of deliveries per canister, content per spray, and content uniformity

Single actuation of MDI formulation was sprayed on a dark surface, and the time taken to disappear was measured. The canister was exhausted completely and noted the total number of actuations. The amount of drug delivered per actuation was measured by the HPLC method. Initially, six actuations were done in an Eppendorf tube and allowed to evaporate the solvent. The canister was shaken thoroughly with a 5 s gap between each spray. After evaporation, the residue was dispersed by vortexing into 1 ml methanol, followed by centrifugation at 1000 rpm. 20 μl of supernatant was injected into an analytical HPLC and estimated UA and OA at 210 nm [13]. An Agilent 1260 Infinity (Agilent Technology, USA) instrument was used for this estimation, equipped with a degasser and a flexible pump (quaternary UHPLC pump). The maximum pressure was fixed at 150 bar. Phenomenex Luna® C_18_ HPLC column (250 x 4.6 mm, 5 μm) was used to separate UA and OA. The detection wavelength was set at 210 nm. Column temperature and flow rate were maintained at 25°C and 1.0 ml/min, respectively. For content uniformity, the variation was checked at the spray of 1^st^, 20^th^, 40^th^, 60^th^, 80^th^, and 100^th^ spray from MDI. The single spray was done in an Eppendorf tube. UA and OA content uniformity was measured using the method described in the content per spray section.

#### Assessment of aerodynamic particle size

Initially, an inhaler pump (S1 Fig) was fabricated using a 3D printer machine (Ultimaker^3^, Utrecht, Netherlands; Software-CATIA-V5). Further, an eight-stage Andersen cascade impactor measured the aerodynamic particle size distribution of UA and OA MDI formulation. The formulations were sprayed three times into this impactor under a flow rate of 28.3 L/min. Depending upon their sizes, scattered particles were deposited at impactor stages. The deposited UA and OA were rinsed from the impactor compartment with methanol. The samples were analyzed using HPLC. Later, these values were projected into the MMADCALCULATOR to calculate the Median Mass Aerodynamic Diameter (MMAD) and geometric standard deviation (GSD) [14]. Particle size <5 μm is known as fine particle fraction, representing a higher probability of deep lung penetration. Particle size <1.0 μm is known as extra fine particles that are helpful to increase drug deposition in the lungs’ periphery [15]. These parameters were assessed to understand the delivery pattern and lung deposition of UA and OA.

### Pharmacokinetic study

#### Animals handling and dose administration

A single-dose pharmacokinetic study was performed on 6–8 week, non-anesthetized old female Wistar rats (n = 6) with an average body weight of 250±50 g. This animal experimentation was performed at the ICMR-National Institute for Research in Reproductive Health (ICMR-NIRRH), Mumbai, India (equipped with advanced infrastructure for animal handling and specialized staff and research scientist) with the permission of Institute Animal Ethical Committee (IAEC no: 1/19). Animals were kept in a controlled environment at a temperature (23±1°C) and humidity (55±5%) with a 14 h light/10 h dark cycle. Soy-free in-house prepared pallets and sterilized water were provided to these animals. The experiments were performed as per the guidelines of the Committee for Control and Supervision of Experimental Animals (CPCSEA), India. A combination of UA (120 μg) and OA (120 μg) was administered in a single actuator to six rats to study the pharmacokinetic parameters in the form of MDI. After a single dose of administration, blood samples were withdrawn from the retro-orbital plexus at a specified time (0 to 24 h). All these animals were found to be in good health after the experimentation, and none were euthanized.

#### Estimation of UA and OA in rat plasma

Quadrupole time of flight (Q-TOF) LCMS (Agilent iFunnel technology; 6550) with Syncronis™ C-18 Columns (Thermo Scientific, 3 μm, 100 x 2.1 mm) was used to quantify the selected drugs in plasma samples. The LCMS instrument was equipped with a PDA detector. After preparing the standard stock solution, serial dilution was made to prepare 25–400 ng/ml reference stock solution. 20 μl of glycyrrhetinic acid was added as an internal standard. In addition, a blank plasma sample was also added to each reference solution. After filtering through a 0.22 μm nylon filter, these reference stock solutions were injected into Q-TOF LCMS. The mobile phase was contained 20mM ammonium acetate buffer (pH-5.0) containing 0.1% v/v formic acid-acetonitrile [16]. A gradient flow system was adopted for better separation of these drugs.

#### Sample preparation and extraction of UA and OA from rat plasma

A stock solution was prepared for UA and OA individually by dissolving 1.0 mg drug in 1.0 ml of methanol. Serial dilution was made to make a final standard solution of 100, 200, 400, 800, and 1000 ng/ml. Further, a combined diluted solution was prepared for estimating UA and OA simultaneously. 500 μl of plasma sample was transferred to a disposable Eppendorf tube. 300 μl of MTBE was added to each Eppendorf tube, followed by 5 min vortexing using Eppendorf ThermoMixer® F. The resultant solution was centrifuged at 2000g for 10 min. 200 μl of the upper organic layer was collected to a new Eppendorf tube and allowed to dry using Eppendorf ThermoMixer® F at 45°C with continuous shaking. The residue was stored at 4°C till further analysis. At the analysis time, the thawed residue was reconstituted with 1000 μl of mobile phase followed by centrifugation at 10000 g for 10 min. 500 μl of the above sample was transferred to a glass vial, and an aliquot of 5.0 μl was analyzed using LCMS.

#### Pharmacokinetic assessment

Blood samples were withdrawn from the retro-orbital plexus at 0, 1, 4, 6, 8, 12, and 24 h after a single dose of administration. UA and OA were extracted as mentioned previously, and estimated their concentration using LCMS. The peak plasma concentration (C_max_) and time (T_max_) required to reach C_max_ were obtained through experimental observations. The elimination rate constant was determined from the slope of the terminal elimination phase plotted between log concentration vs. time curve. Using the trapezoidal rule, the area under the curve (AUC_0-24_) was calculated from the plasma concentration vs. time (up to 24 h) plot. The extrapolated area (24 h to ∞) was calculated by dividing the final measured concentration by the constant elimination rate. AUC_0-∞_ was calculated as a sum area from 0 to ∞.

### Acute toxicity study

The acute toxicity (OECD guideline 425) was performed with the prior permission of IAEC (IAEC no: 1/19) and was executed at the same premises. A total of 12 rats were divided into two groups: control (blank propellant) and treatment (UA (120 μg) and OA (120 μg)). Each group contained three male and three female rats. The vehicle control animals received blank propellant, whereas the treatment groups’ animals received UA and OA through MDI. The rats were single-dose MDI (acute toxicity) and were monitored for clinical signs of toxicity or behavior for 1 h intervals for 4 h and then 4 h intervals for 24 h. These rats were further kept under observation for once a day up to 14 days. Weekly body weight and feed weight were observed. On the 15^th^ day, blood was collected for hematological and biochemical analysis. Under ethical permission, these experimental animals were euthanized by CO_2_ asphyxiation. All the vital organs were carefully removed, trimmed to remove excess fats, and recorded weight. These organs were fixed in 10% neutral buffered formalin (NBF) for histopathological analysis.

### Statistical analysis

Parametric data of the biochemical, hematological, and relative organ weight analyses were expressed as mean ± SD. The Student t-test was conducted to establish the significant difference between control and treatment groups in acute toxicity data and anti-tuberculosis activity. One-way ANOVA was used to establish the considerable difference. The values were considered statistically significant at a level of P ≤ 0.05.

## Results

### Anti-tuberculosis activity in-vitro using H_37_R_v_ strains

**Fig 1:**
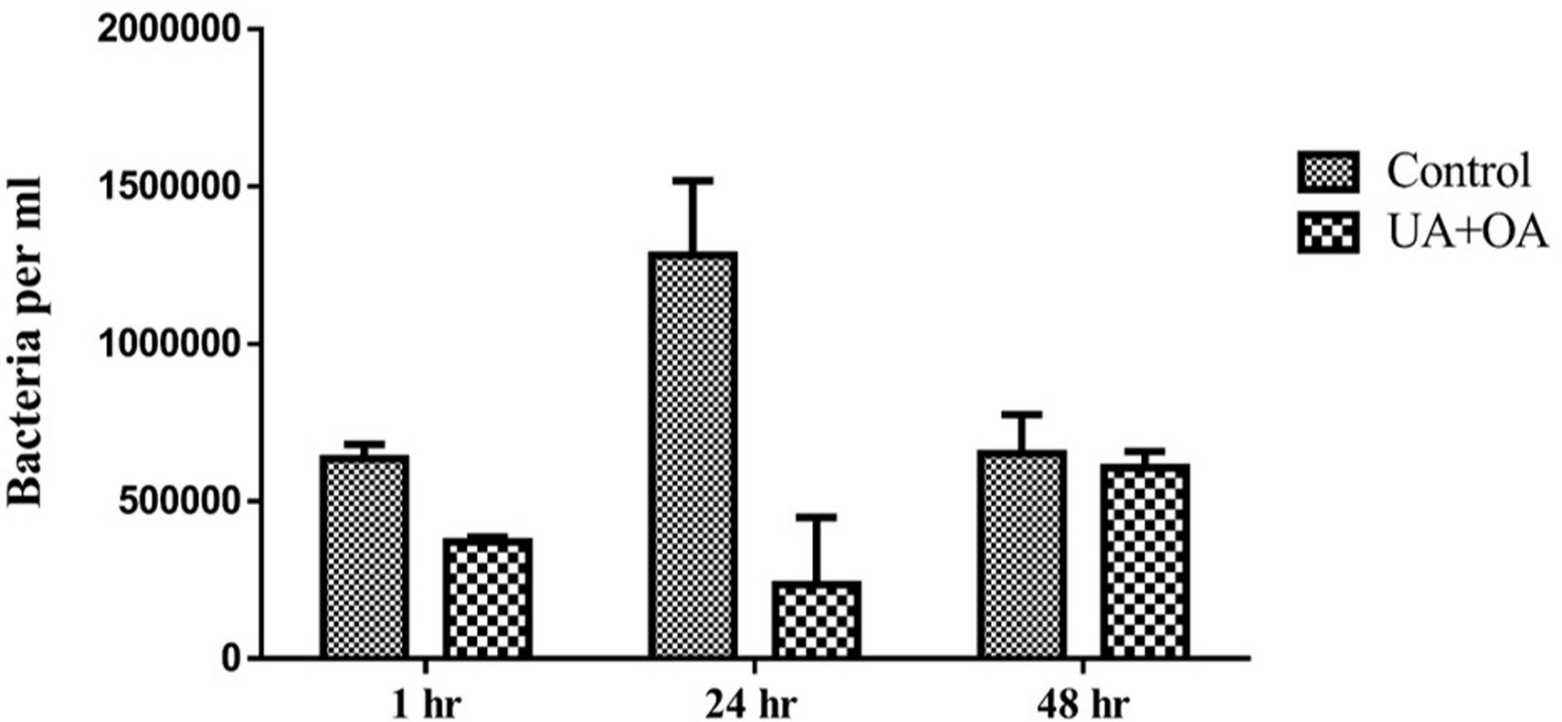
*In-vitro* anti-tuberculosis activity of UA and OA using H_37_Rv strain. MH-S cells infected with the H_37_Rv strain showed lower bacterial load at 1 and 24 h when treated with UA and OA compared to the control (Isoniazid treated). The values are expressed as mean ± SD, n = 3.

In this study, the MH-S cells infected with the H_37_Rv strain showed a reduction in bacterial load in UA and OA-treated cells compared to the control (isoniazid treated) at 24 h (Fig 1). The MH-S cells showed a slight decrease in bacterial load at one hour. However, the bacterial load was reduced ten times by 24 h in UA and OA-treated cells compared to the control cells. No difference in the bacterial load was observed at 48 h reading in the UA and OA cells compared to the control.

### Characterization of MDI

Prepared MDI was characterized for different *in-vitro* characterization. The prepared formulations showed the desired vapor pressure of 70–75 psi. No additional solvent/co-solvent was used to dissolve UA and OA.

#### Average weight per metered dose and number of deliveries per canister

The fabricated 3D model inhaler pump successfully delivered the metered dose. The metering valve accurately determined the metered dose. In every five sprays, the weight of the canister was recorded. The average weight per metered dose was found as 58.2±1.54 mg. The canister was capped and sealed correctly and had no leakage. Leakage can interfere with actuation, resulting in incomplete actuation. The canister was filled with 100 metered-dose, and the no of actuations was obtained as 102±3. It signifies that the valve of the canister is functioning correctly.

#### Rate of evaporation, content per spray, content uniformity, and average weight per actuation

The prepared formulation showed an excellent spray pattern. The evaporation rate is an important measure that helps predict the respiratory tract’s spray pattern and drug deposition. UA and OA were dissolved in the propellant without any solvent and co-solvent. The time it took to evaporate one spray was less than 5 s. The delivery dose was checked by actuating the valve six times and estimating the content using the HPLC. OA was eluted first with a retention time of 4.98 ± 0.04 min, followed by UA with RT 5.17±0.02 min. The content per spray is essential to deliver a uniform dose in each actuation. The average content of UA and OA per actuation was 100.76±1.44% and 101.08±1.42%, respectively. The content uniformity was performed at the spray of 1^st^, 20^th^, 40^th^, 60^th^, 80^th^, and 100^th^ actuation. The recorded result was satisfactory as the average spray content per actuation was 100.29±1.43% for UA and 99.73±1.28% for OA. Therefore, both the drugs’ content uniformity was maintained until the last actuator of the canister.

#### Aerodynamic particle size distribution

The MMAD is the critical constraint that regulates the sedimentation of inhaled particles in the different regions of the lungs. Particle size range of 0.5–5 μm can travel to the alveoli region. The deposition pattern of the prepared MDI formulation was quantified by the content of particle deposition in each stage of the cascade impactor. Fine particle fraction, extra-fine particle fraction, and the emitted dose were found as 20.91±3.65%, 12.47±3.16%, and 72.48±7.89%, respectively. Aerodynamic particle size was obtained as 4.44±0.74 μm with a geometric standard deviation of 3.66±2.93. That data supported that prepared MDI formulation can reach deeper into the lungs.

### Pharmacokinetic study

#### Estimation of UA and OA in rat plasma administered through MDI

Q-TOF LC-MS was employed here in the simultaneous estimation of UA and OA. The electron spray ionization technique showed better sensitivity and linearity by producing high voltage to generate ions to the methanolic solution of UA and OA. The molecular mass of UA and OA was detected at 455.35 *m/z*. OA was eluted first, followed by UA with a retention time of 16.707±0.06 min and 16.809±0.04 min, respectively. The internal standard peak was detected at 15.97±0.33 min with a mass peak of 469.33 *m/z*.

#### Pharmacokinetic assessment

**Fig 2:**
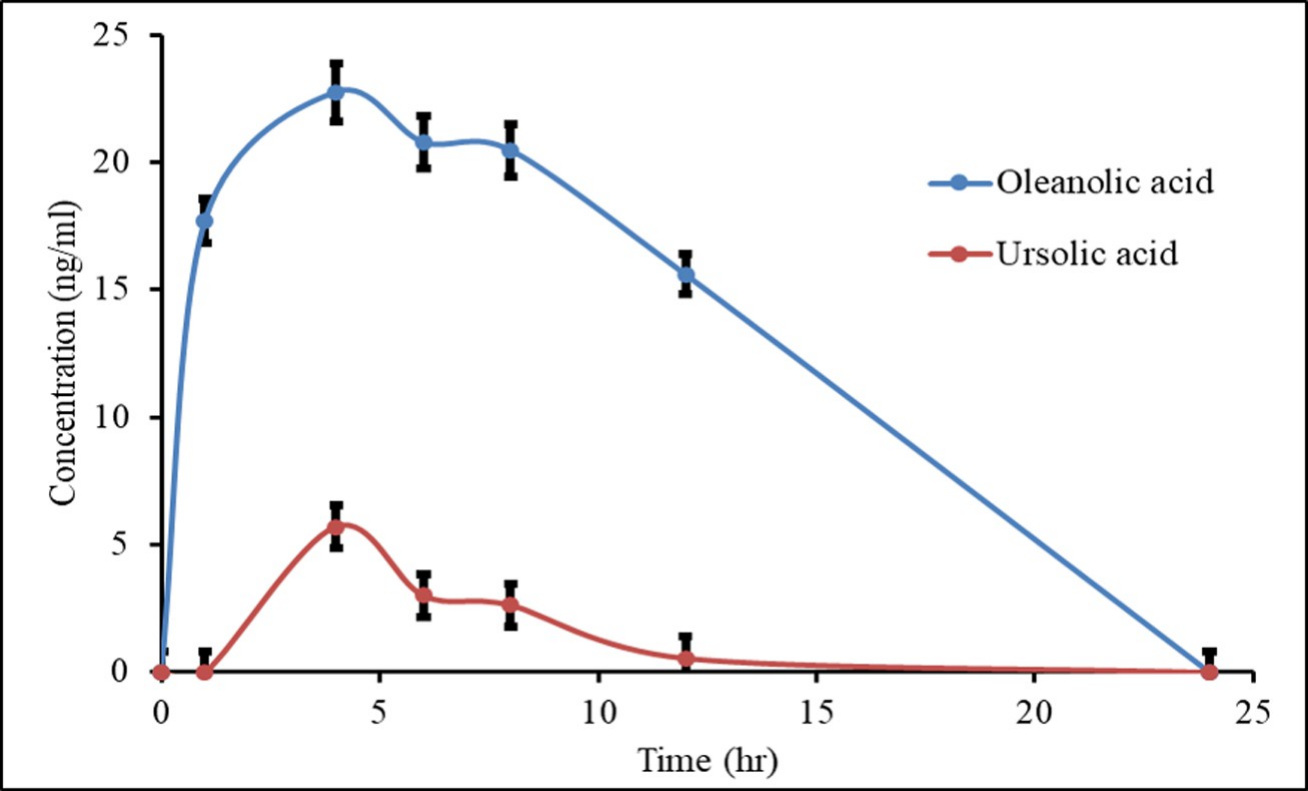
Concentration vs. time profile of UA and OA. Both drugs showed maximum plasma concentration at 4 h and could not detect at 24 hr in plasma.

A single actuator containing 240 μg of UA and OA in combination (UA and OA 120 μg each) was administered to experimental rats. The plasma drug concentration was determined for 24 h at different time points. The biological half-life of UA and OA was 9.23±0.104 and 8.93±0.166 h, respectively (Table 1). C_max_ was 5.70±0.173 ng/ml for UA and 22.75±0.851 ng/ml for OA; furthermore, the T_max_ of both materials was found as 4 h. The plasma concentration vs. time profile has been depicted in (Fig 2).

**Table 1:** Pharmacokinetics parameters of UA and OA administered through MDI (240 μg/kg BW) in Wistar rats.

Drug	C_max_ (ng/ml)	T_max_ (hr)	AUC (ng*hr/ml)	Half-life (hr)	Elimination rate constant (hr^-1^)
UA	5.70 ± 0.173	4±0.00	26.48±1.29	9.23±0.104	0.075±0.002
OA	22.75±0.851	4±0.00	428.25±1.87	8.93±0.166	0.077±0.002

The data presented here in mean ± SD, n = 6

### Acute toxicity study

The female Wistar rats were exposed to OA and UA through MDI. This 14-day acute toxicity study did not indicate any death or adverse effects. The treatment group rats did not demonstrate abnormal behavior due to the metered-dose treatment. No toxic evidence of toxicity was observed in the gross examination of these female rats during the necropsy of treatment and control groups.

#### Effect on body weight and organ weight

**Fig 3:**
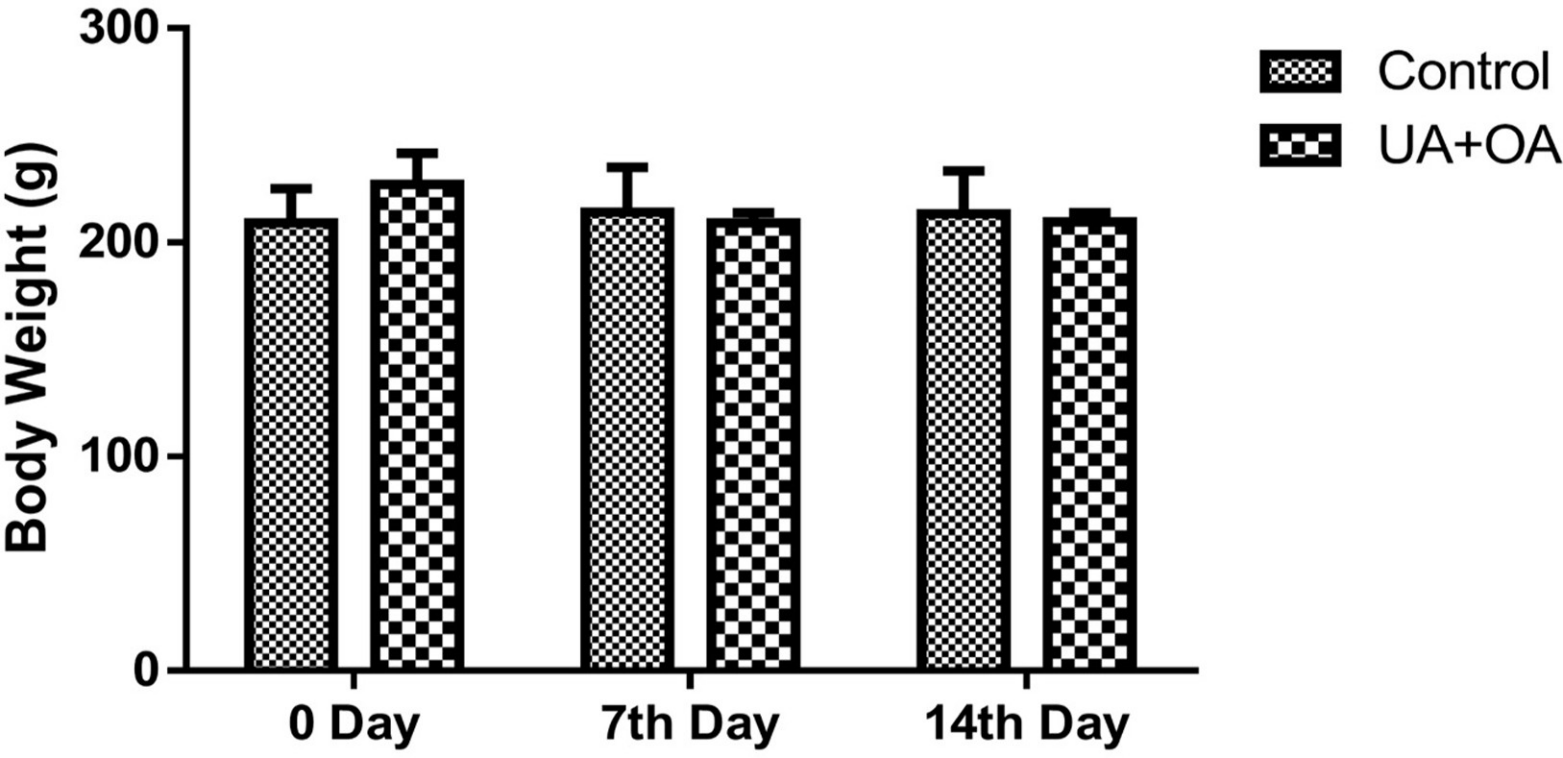
Changes in body weight during acute toxicity study. No significant differences were detected between the treatment group and the control group.

The animals were monitored weekly for changes in body weight for up to 14 days. Compared to the control group, no changes in body weight were observed in female rats exposed to metered-dose of UA and OA (Fig 3).

These animals were sacrificed on the 14^th^ day and recorded organ weight. The treatment did not show any changes in the organ weight compared to the vehicle control (Table 2).

**Table 2:** Changes in organ weight of female rats of the control group in an acute toxicity.

Organ	Control	MDI
Liver	3.137±0.22	3.23±0.11
Kidney	0.764±0.06	0.797±0.04
Brain	0.830±0.06	0.830±0.03
Heart	0.518±0.12	0.514±0.04
Spleen	0.198±0.05	0.180±0.01
Lungs	0.612±0.87	0.654±0.09
Trachea	0.110±0.04	0.128±0.06
Uterus	0.187±0.02	0.274±0.10
Adrenal Gland	0.030±0.01	0.034±0.004
Ovaries	0.056±0.005	0.059±0.01

The values are expressed as mean±SD, n = 6.

#### Effect on serum biochemistry and hematology

The biochemical parameters of the treatment groups were compared with the control groups. The effect of UA and OA on serum biochemistry in female rats showed no changes in the parameters tested (Table 3), except with the serum calcium levels, which were increased slightly in the treatment group compared to the control.

**Table 3:** Effect of MDI on serum biochemical parameters of female rats in acute toxicity.

Parameter	Control	MDI
SGPT (IU/L)	55.10±9.99	57.38±12.96
SGOT (IU/L)	149.1±18.12	132.4±14.22
Alkaline phosphatase (IU/L)	228.0±340.0	209.0±631.0
Phosphate (mg/dl)	5.915±0.83	5.703±0.36
Direct bilirubin (mg/dl)	0.045±0.005	0.042±0.012
Total bilirubin (mg/dl)	0.085±0.019	0.078±0.024
Urea (mg/dl)	30.880±2.32	31.97±5.69
Creatinine (mg/dl)	0.568±0.042	0.593±0.030
Glucose (mg/dl)	116.800±21.32	119.1±5.83
Triglycerides (mg/dl)	58.67±14.92	67.33±9.87
Cholesterol (mg/dl)	93.33±13.54	99.17±7.14
HDLC (mg/dl)	39.53±6.62	43.97±7.39
LDL (mg/dl)	17.41±1.98	17.93±1.98
Uric Acid (mg/dl)	1.452±0.30	1.525±0.323
Calcium (mg/dl)	10.58±0.21	10.82±0.23*
Albumin (g/dl)	3.780±0.42	3.945±0.25
Protein (mg/dl)	7.332±0.17	7.870±0.51

The values are expressed as mean±SD; n = 6;

*p≤0.05; SGOT-Serum glutamic oxaloacetic transaminase, SGPT-Serum glutamic pyruvic transaminase, HDLC-High density lipoprotein cholesterol, LDL-Low density lipoprotein.

The hematological blood parameters were compared with the control (Table 4). Only changes were observed in the mean corpuscular hemoglobin concentration, which was increased after acute exposure to MDI in female rats.

**Table 4:** Effect of MDI on hematological parameters of female rats in the acute toxicity.

Parameter	Control	MDI
Hemoglobin (g %)	16.92±0.56	16.58±0.81
RBC (x 10^6^ / cmm)	8.63±0.32	8.41±0.40
WBC (x 10^3^ / cmm)	8.33±3.54	6.520±0.66
Platelets (x 10^3^ / cmm)	275.8±244.6	217.2±62.30
Packed cell volume (%)	49.78±1.73	47.30±2.090
MCV (fl)	57.75±1.08	56.32±1.81
MCH(pg)	19.55±0.43	19.68±0.944
MCHC (g/dl)	33.92±0.34	35.02±0.68**
Neutrophils (%)	37.67±6.74	42.00±3.16
Eosinophils (%)	2.17±0.98	2.400±1.52
Lymphocytes (%)	59.17±6.88	55.00±4.18
Monocytes (%)	1.00±0.63	0.60±0.55

The values are expressed as mean±SD, n = 6,

**p≤0.01; RBC-Red blood corpuscles, WBC-White blood corpuscle, MCV- Mean corpuscular volume; MCH- Mean corpuscular hemoglobin; MCHC- Mean corpuscular hemoglobin concentration.

#### Effect on histopathology of the vital organs

**Fig 4:**
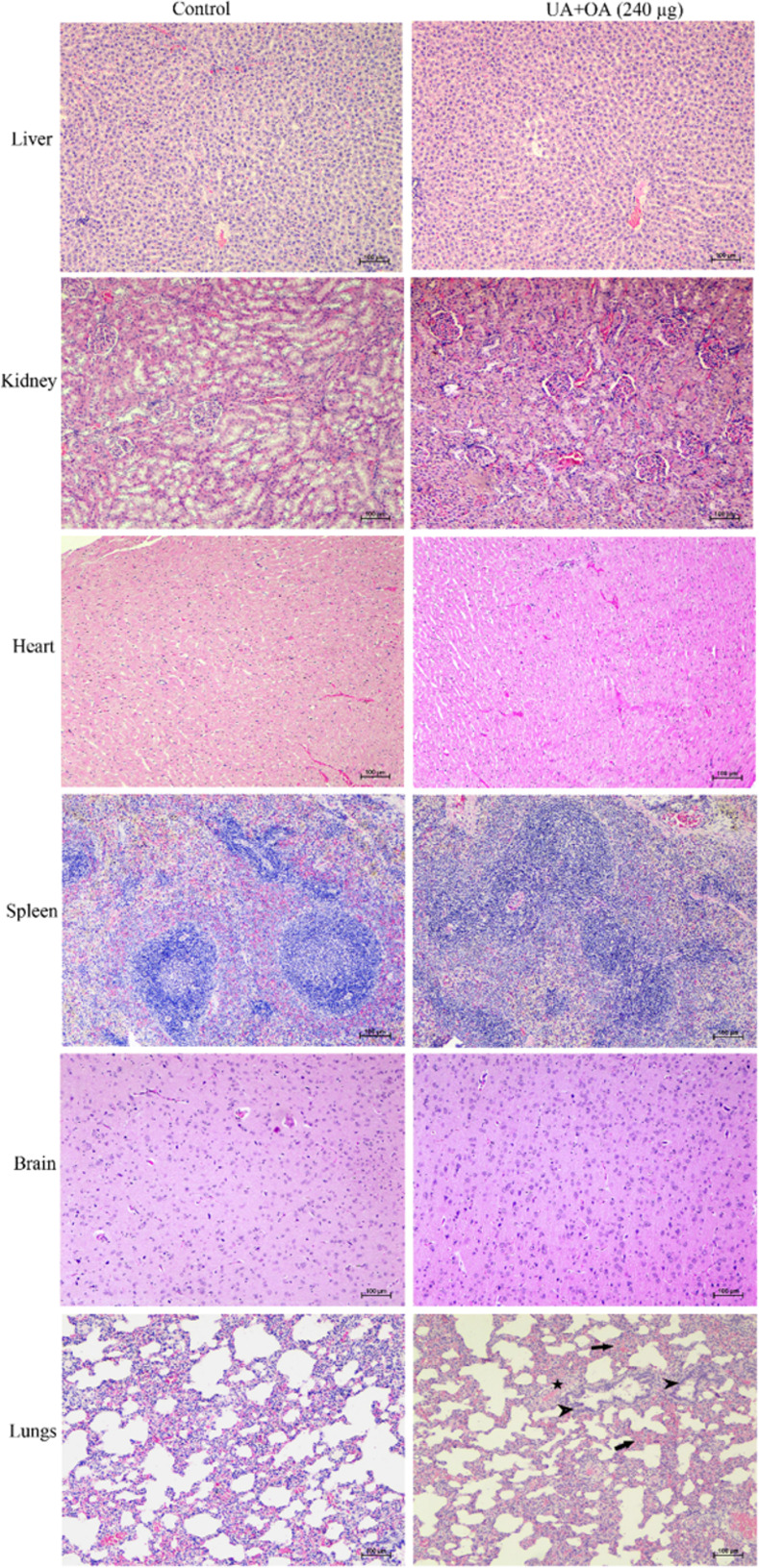
Effect of combined UA and OA exposure on histopathology of the vital organ in acute toxicity through MDI. The perturbations in the pathology of the lungs of the exposed rats have been marked with arrowhead (Magnification 10 X).

The histopathological changes were marked in the MDI-exposed female rats in the acute exposure compared with the control group rats. No significant histopathological alterations in vital organs were observed in female rats exposed to MDI compared to the control. However, lung tissue showed congestions and hemorrhagic lesions in the lung parenchyma with distorted alveolar tissue and peribronchiolar aggregation of MNCs in the MDI-exposed animals. Furthermore, focal infiltration of mononuclear cells in the interstitial space between alveoli and bronchiolar epithelial degeneration was observed in the MDI-exposed animals compared to the control (Fig 4).

## Discussion

It is well established that many plant species biosynthesize UA and OA along with other Triterpenes. Hence, most of the light has been focused on extracting these triterpenoids and then studying their effect on humans. The pharmacological effects of UA and OA are well established in several diseases like anti-diabetic, anti-tumor, antibacterial, anti-inflammatory, and hepato-protective. The present study showed the mixture of UA and OA at a 1:1 ratio had a synergistic effect on the anti-tuberculosis activity of H_37_Rv in MH-S cells. This effect at lower concentrations also reported earlier [5], and our study is also supported the same.

For the effective management of tuberculosis, drug targeting the lungs is a prerequisite as the lung is mainly affected due to tuberculosis. MDI formulation was prepared to achieve this goal by incorporating UA and OA. As both these drugs dissolved in the hydrofluoroalkane (selected propellant), no additional solvent or co-solvent need in this formulation design. Therefore, an excellent rate of evaporation was observed after the spray. The recorded pressure of 70–75 psi inside the canister is satisfactory for delivering the uniform dose of UA and OA in each delivery. Additionally, the dose uniformity persisted until the canister’s last prayer.

After single actuation, this MDI formulation was administered to female Wister rats. At specific time intervals, the blood samples were collected from the retro-orbital vein of rats. After extracting the drug from the plasma, these drugs were estimated using the LSMS technique. The biological half-life of UA and OA was 9.23±0.104 h and 8.93±0.166 h, respectively. Due to the co-administration of UA and OA, the biological half-life of these drugs increased, signifying the longer persistence of these drugs in the systemic circulation. The C_max_ values were also considerably low, indicating that the lungs’ drug concentration is more than the systemic availability.

An acute toxicity study was performed here to understand the adverse effects of these drugs during the two weeks of inhalation exposure. Treatment of rats with OA and UA was found safe at the doses tested in an acute toxicity study [8]. In this study, no adverse effect of inhalation exposure was observed on rats. Changes in organ weight or body weight represent drug-associated toxic effects. No effect on body weight changes has been reported in mice treated with UA (100 μg/kg BW) and OA (200 μg/kg BW) through a subcutaneous route for 11 weeks [17]. In this study, no detectable body or organ weights were observed, altering the dose and route of administration. This study demonstrates the inhalation exposure of UA and OA through intra-tracheal route administration using MDI is safe as this exposure did not possess any toxic effects on the organs. Serum biochemistry parameters are critical in evaluating the harmful effects of the given chemical on general health status as well effect on metabolic processes. There is no drastic change in the biochemical and hematological parameters in the presented study. Slight changes were observed in the mean corpuscular hemoglobin concentration. However, there is no significant impact observed on overall animal health.

## Conclusion

Tuberculosis is a lethal disease accounting for 18% of deaths in India annually. The localized treatment can serve as an emerging approach for better management of tuberculosis. After confirming the *in-vitro* antitubercular activity, metered dose inhaler was formulated by incorporating UA and OA for targeted delivery to the lung. Prepared MDI showed good spray pattern and dose uniformity until the canister’s last actuation. During the *in-vitro* and *in-vivo* studies, the fabricated device efficiently delivered metered doses in characterization and animal experimentation. This effective delivery is a holistic approach to establishing an anti-TB effect of UA-OA (1:1) along with its detailed toxicity profile *in vivo*. This is the first report of an acute toxicity study of metered-dose of UA and OA and their pharmacokinetic Assessment in female Wistar rats. Further investigations are required to evaluate the efficacy of UA and OA in combination in an *in-vivo* TB model.

## Supporting information

S1 Fig Fabricated inhaler pump using a 3D printer machine for animal administration. (TIF)
