# Supplementary figures and images for: Targeted delivery of ursolic acid and oleanolic acid to lungs in the form of an inhaler for the management of tuberculosis: Pharmacokinetic and toxicity assessment
Source: PLoS One. 2022 Dec 29;17(12):e0278103. doi: 10.1371/journal.pone.0278103 (PMC9799288; doi:10.1371/journal.pone.0278103)

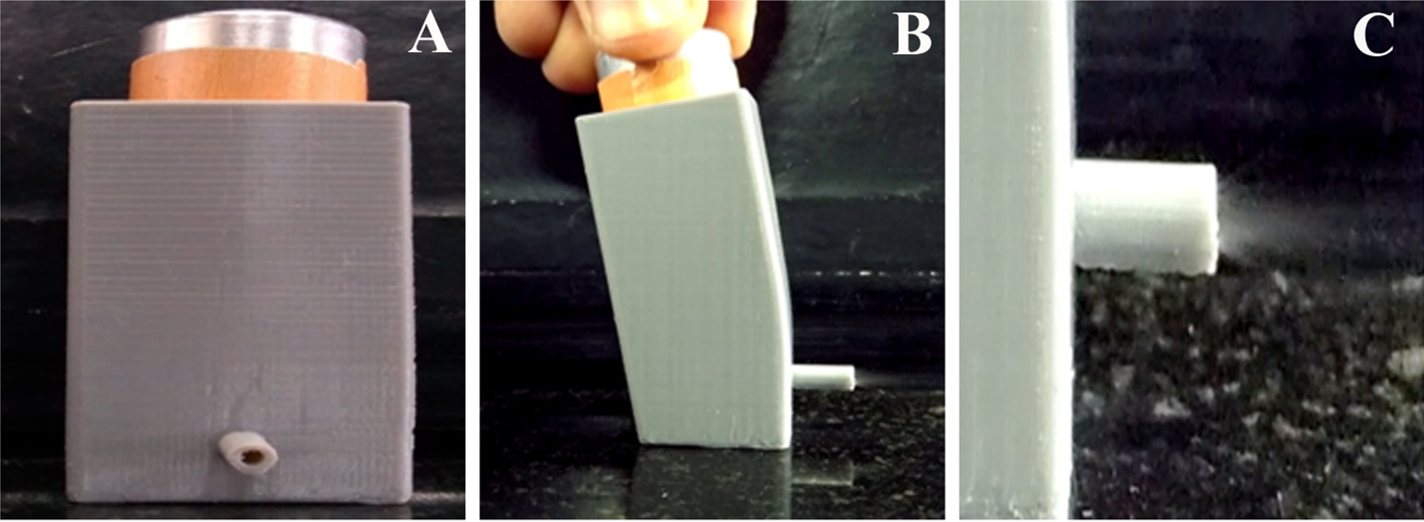

Supplement: S1 Fig — (TIF) [file pone.0278103.s001.tif]
